# Factors of Nurses' Using Mobile Applications to Provide Home Nursing Care: A Social Cognitive Theory Perspective

**DOI:** 10.1155/jonm/4002293

**Published:** 2025-06-18

**Authors:** Jing Cheng, Tailai Wu, Kangni Ren, Zhaohua Deng

**Affiliations:** ^1^Outpatient Department Office, Tongji Hospital, Tongji Medical College, Huazhong University of Science and Technology, Wuhan, China; ^2^Department of Management Science and Engineering, School of Management, Wuhan University of Technology, Wuhan, China; ^3^School of Medicine and Health Management, Tongji Medical College, Huazhong University of Science and Technology, Wuhan, China; ^4^Yunnan Key Laboratory of Service Computing, Yunnan University of Finance and Economics, Kunming, China; ^5^School of Management, Huazhong University of Science and Technology, Wuhan, China

**Keywords:** mobile home nursing care applications, outcome expectations, self-efficacy, social cognitive theory

## Abstract

**Background:** The aging of global population, coupled with the increase in chronic noncommunicable diseases, has made home nursing care services an increasingly vital component of elderly care. Thus, the demand for such services has increased, prompting the development of mobile home nursing care applications (MHNCAs) to assist nurses in delivering home-based care. However, nurses' willingness to use MHNCAs remains relatively low.

**Aim:** This study aims to investigate what and how the factors affect nurses' use of MHNCAs.

**Methods:** Based on social cognitive theory, we have formulated a research model to identify and explain the relationships between factors and nurses' use of MHNCAs. We use a multiple analytical approach including structural equation modeling (SEM) and artificial neural network (ANN) to examine the research model.

**Results:** The SEM analysis results show that environmental factors could affect nurses' use of MHNCAs through personal factors, while the ANN analysis results highlight outcome expectations as the strongest predictor of nurses' MHNCA usage.

**Conclusions:** The factors and their working mechanisms of nurses' using MHNCAs are uncovered and validated. Our study explores novel factors of using of mobile health applications from the perspective of nurses and novel mobile applications. Besides, we contextualize and extend social cognitive theory within our study. Moreover, we unveil the underlying mechanisms by confirming the mediating roles of self-efficacy and outcome expectations.

**Implications for Nursing Management:** Our findings can benefit nursing managers, nursing educators, and the broader nursing community by informing the design of MHNCAs tailored for nurses, supporting the uptake of MHNCAs, and enhancing the training and education related to nurses' technology use. Therefore, our study underscores the cutting-edge advancements in mobile health applications within the digital realm and the nursing context, highlighting the importance of these technologies in contemporary nursing practices.

## 1. Introduction

With global population aging, the need for home care services is increasing. People aged over 60 will constitute more than 15% of the world's population by 2030, and this figure is projected to surge to 2.1 billion by 2050 [1]. 1 Meanwhile, the most common health conditions for elderly people are chronic noncommunicable diseases such as diabetes, chronic obstructive pulmonary disease, or dementia [1]. To promote healthy aging, home care services should be provided for older adults [2]. Home care services have been shown to be associated with the reduction of healthcare costs, readmission rates, and death rates [3]. In the United States, the total expenditure for home care services reached 113.5 billion US dollars in 2020 [4], while more than 80% of older adults are expected to receive home care services in their community in 2018 in China [5]. Several European countries have a similar trend as well [6]. Among the specific home care services, nursing care is a significant service needed by older adults [7].

Home nursing care services provide disease diagnosis and prevention, medication administration, patient education, emotional support, and collaboration with other healthcare professionals [8]. Home nursing care could reduce admissions and readmission rates,2 hospital stay lengths, and hospitalization costs [9–11]. Although the need for home nursing care is huge, there is still a shortage of nursing services, and this shortage has been intensified during the period of the pandemic [12, 13]. To alleviate this shortage, several mobile home nursing care applications (MHNCAs) have emerged to facilitate the provision of nursing services. For example, Go2Nurse, a US mobile nursing application, could help users connect with licensed nurses easily and ask nurses to provide home care [14]. Gold Nurse, a Chinese mobile nursing application, operates in 400 Chinese cities with almost 200 thousand nurses to provide home nursing care services. 3 These MHNCAs operate on mobile devices and serve as mobile internet-based platforms, allowing patients and nurses to interact and transact with each other. Thus, patients' home nursing care needs could be satisfied by using MHNCAs [15].

However, nurses' willingness to use MHNCAs to provide home care nursing services is still not high enough [16]. Thus, current MHNCAs are not playing their full role in alleviating the need for home nursing care and the health conditions of older adults who need home nursing care would be worsened [17, 18]. Thus, it is necessary to promote the provision and quality of home nursing care by motivating nurses' use of MHNCAs. Besides, since MHNCAs could be used by nurses for providing home nursing care, they are different from traditional mobile health applications which are used by patients and nursing information systems, which focus on in-hospital clinical service in terms of users and types. This uniqueness would provoke novel factors that have not been examined before. Therefore, the factors of nurses' use of MHNCAs are necessary to be explored to promote home nursing care provision.

To explore the factors of nurses' use of MHNCAs, we first examine the literature on mobile health application use since MHNCAs are related to mobile health applications. Previous literature on mobile health applications has focused more on the perspectives of patients or consumers than on those of health professionals [19–21]. Therefore, factors explored in the previous literature on mobile health application use may not reflect the characteristics of nurses. As MHNCAs are part of the information systems used by nurses, we also reviewed the literature on the use of nursing information systems and the specific use of mobile nursing information systems. We found that the previous literature on these systems has been used for clinical nursing services, not for home nursing care services [22–27]. Therefore, nursing information systems and mobile nursing information systems in the previous literature have different functions and different roles for nurses, which are different from MHNCAs. Hence, the factors explored in the literature on the use of nursing information systems and mobile nursing information systems do not reflect the characteristics of MHNCAs.

By following the principles from Alvesson and Sandberg [28], we problematize our research question to enhance the contribution of our study. First, we identify and find the assumption of previous literature that only considers the generic factors of mobile health applications from consumers' perspectives or nursing information systems for clinical purposes. Second, we think this assumption is worth to be challenged since generic factors may not capture the context of nurses' use of MHNCAs adequately and could not provide effective practical guidelines for nurse managers to promote the use of MHNCAs and design for nurses. Third, we challenge the assumptions of previous literature by understanding a novel kind of mobile health applications from the perspective of nurses. The novel mobile health applications and nurses' perspectives could bring novel knowledge including novel and contextual factors to relevant literature. Fourth, both theoretical and practical implications could be provided to both researchers and nurse managers. By summarizing the above problematization and considering the practical importance of MHNCAs, we propose our research questions as follows:

  What and how factors affect nurses' use of MHNCAs to provide home nursing care services?

To solve the above research questions, we establish a research model based on social cognitive theory. This model is rigorously validated through a two-wave survey, targeting nurses with experience on using MHNCAs. By leveraging both structural equation modeling (SEM) and artificial neural network (ANN) techniques, we not only examine the hypothesized relationships in our research model but also explore the importance of factors. Based on these results, the implications, limitations, and future directions of our study are discussed.

Our study makes three main contributions: First, novel factors of mobile health application use are explored from the novel mobile applications and nurses' perspectives. Compared with the previous literature on mobile health applications for consumers and on information systems for clinical services in-hospital, our study investigates the factors of nurses' use of MHNCAs, which connect nurses with patients online. Novel and contextual factors that reflect the characteristics of nurses and home nursing care are explored in this study. Besides, the differential effects of these novel factors on nurses' use of MHNCAs are also identified in this paper. Second, social cognitive theory is developed by being contextualized and extended in our study. To deepen our understanding of this topic, we not only extend social cognitive theory into our context as the overarching theory to provide a complete view but also contextualize this theory by decomposing environmental and personal factors. Besides, the validation of the application of social cognitive theory in our study could imply that social cognitive theory could be generalized to the nursing context and the application scope of social cognitive theory is enlarged. Therefore, we make a significant theoretical contribution by testing social cognitive theory in a new context and adding novel factors to develop this theory. Third, the underlying mechanisms are revealed by confirming the mediating roles of self-efficacy and outcome expectations. To be specific, the full and partial mediating roles of self-efficacy and outcome expectations are uncovered. Then, the underlying mechanisms of our proposed relationships are captured, which facilitates the theoretical understanding of nurses' use of MHNCAs.

### 1.1. Literature Review

Our study involves three main streams of literature: mobile health application use, nursing information system use, and mobile nursing information system use. We review these streams in the following sections.

#### 1.1.1. Mobile Health Application Use

Mobile health applications are applications embedded on mobile devices to provide health information and support medical activities [29]. Mobile health applications studied in previous literature include fitness apps, clinical support apps, and mobile health services [30–32]. Many of them are designed and provided for patients or consumers. Several theories have served as theoretical foundations in previous literature, including motivational theory [19], protection motivation theory and social cognitive theory [33], and the extended unified theory of acceptance and use of technology [34, 35]. Therefore, previous literature has explored social, psychological, and technological factors. However, these factors were identified from the patient or consumer perspective, failing to capture the characteristics of nurses, such as job requirements or hospital norms.

#### 1.1.2. Nursing Information System Use

The second stream is the literature on factors of using nursing information systems, which are used by nurses to provide health care services [36]. Many nursing information systems have been studied, including electronic health records, sensor-based medication systems, and care plan systems [22, 24, 25]. To predict nurses' use of nursing information systems, several information technology adoption theories have been applied in previous studies, including the technology acceptance model, the theory of planned behavior, and the unified theory of acceptance and use of technology [25, 37–39]. Therefore, some technical, social, and individual factors have been studied. However, previous literature on nursing information systems has mainly focused on their application within hospital settings for clinical care and not considered the characteristics of MHNCAs, which facilitate nurse–patient interactions in the home context. Besides, since prior literature on nursing information system use has paid little attention to the mobile context, we further review the literature on mobile nursing information system use.

#### 1.1.3. Mobile Nursing Information System Use

Mobile nursing information systems are the information systems that nurses can use to provide care services anywhere and anytime [40]. Several mobile nursing information systems have been investigated, including mobile electronic medical records, personal digital assistants, and mobile e-nursing carts [23, 26, 27]. Similar to the studies of nursing information systems, previous literature on mobile nursing information systems also grounded their research on theories such as the technology acceptance model, technology-task fit theory, and innovation diffusion theory [23, 26, 41]. Therefore, several technical and task-related factors are examined. However, many of the mobile nursing information systems examined in existing studies are designed for in-hospital clinical care and do not facilitate the connection between nurses and patients at patients' homes. Therefore, factors identified in mobile nursing information system use cover fewer of the characteristics of home nursing care, which is different from nursing care in hospitals.

To conclude the above sections, although several factors identified in previous literature could be taken as references, the literature on mobile health application use focuses less on the characteristics of nurses, while the literature on nursing information system use and mobile nursing information system use pays insufficient attention to the characteristics of home nursing care. Therefore, factors of nurses' use of MHNCAs should be investigated to enrich relevant literature and promote nurses' use.

### 1.2. Theoretical Foundation

Our study leverages social cognitive theory to explore the antecedents of nurses' use of mobile applications to provide home nursing care services and their underlying mechanisms. The social cognitive theory proposes that people's learning behaviors are determined by the dynamic reciprocal interaction between environmental and personal factors [42]. Environmental factors encompass the physical and social factors that surround individuals, while personal factors concern people's cognitions, personalities, or affections [43]. Among the proposed factors, self-efficacy and outcome expectations are the core constructs that belong to personal factors and could be the primary cognitive forces that guide behaviors [44].

Since MHNCAs provide the platform for interaction between patients and nurses, nurses who have not used MHNCAs could observe the actions of other nurses who used. Therefore, the nurse could make the decision to use MHNCAs by learning from other nurses observationally. Given that nurses' use of MHNCAs may be the result of learning from and observing their peers, this object matches the “what” of social cognitive theory. Besides, social cognitive theory is about the general people, which is not limited to specific social groups and has been applied to study the behaviors of nurses [45, 46]. Thus, the object of our study matches the “who” of social cognitive theory. Therefore, the two similarities between the context of our study and social cognitive theory could support our use of social cognitive theory. Furthermore, previous studies have shown the validity and effectiveness of social cognitive theory in different technology use contexts. For instance, Sun et al. [47] dissected omnichannel service usage by proposing digitalized social cognitive theory; Zheng and Lee [48] explained the negative consequences of problematic IT use based on social cognitive theory. Moreover, social cognitive theory has also been applied in nursing context. For example, Short et al. [49] examined and validated the impact of social cognitive theory constructs on posttreatment breast cancer survivors' physical activity participation. Zhao et al. [50] explored the behaviors and factors of Chinese oncology nurses toward hospice care based on social cognitive theory and found environment and self-efficacy positively associated with hospice care behaviors. Although some classical information systems theories such as technology acceptance model and its extension models have explained use intentions of information systems [51], these theories focus on generic functionalities and ignore other factors of using different information systems. Thus, these classical information systems theories may not help us understand nurses' use of MHNCAs comprehensively and systematically. Therefore, we deem that it is feasible to apply social cognitive theory as the theoretical foundation.

Based on social cognitive theory, environmental and personal factors that influence nurses' use of MHNCAs could be identified. Regarding the environmental factors, since nurses usually belong to medical institutions and possess a strong sense of professional identity [52], we include factors related to medical institutions. Since nurses provide home nursing care services through mobile health applications, factors related to mobile health applications are considered. Moreover, providing home nursing care may influence nurses' work–life balance [53], and social factors are included. Regarding the personal factors, the key roles of self-efficacy and outcome expectations have been shown and we use them as the personal factors [54]. Toward the relationships among factors, we treat personal factors as the underlying mechanisms for the effects of environmental factors on nurses' use of MHNCAs and identify them as the mediators for the above effects. Previous literature also considers the two personal factors as the underlying mechanisms [55, 56]. Our specific theorizing of the above factors is presented in the next section.

### 1.3. Research Model and Hypotheses Development

**Figure 1:**
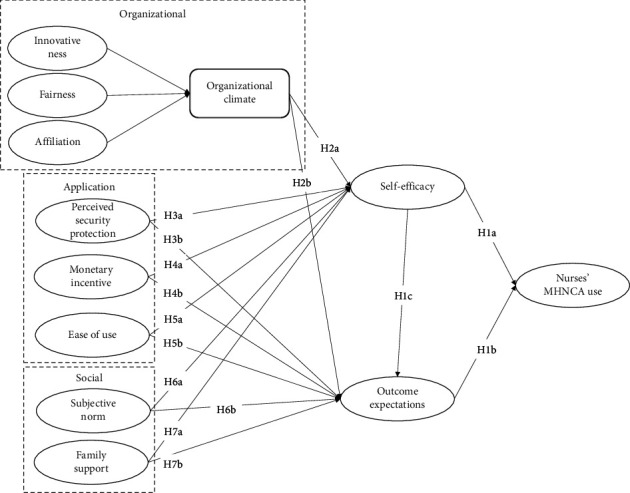
Research model.

Based on social cognitive theory, we propose that nurses' using behavior of MHNCAs is determined by environmental and personal factors [57]. Regarding the environmental factors, we divide them into organizational, application, and social factors since all these factors construct the environment in which nurses use the MHNCAs [58]. Specifically, we consider organizational climate characterized by innovativeness, fairness, and affiliation as the organizational factors [59], while application factors include perceived security protection, monetary incentive, and ease of use [60, 61]. Moreover, social factors could encompass family support and subjective norm [62, 63]. For the personal factors, we use self-efficacy and outcome expectations to represent the personal factors since these two factors have been identified as the critical mechanisms that motivate technology use behaviors [64]. Finally, we assume that environmental factors would impact nurses' using behavior through personal factors. Then, we propose that organizational, application, and social factors influence nurses' use of MHNCAs through their self-efficacy and outcome expectations. The above hypothetical relationships are summarized in our research model depicted in Figure 1.

We select self-efficacy and outcome expectations as the personal factors in this study. Self-efficacy refers to nurses' belief in their capability to use MHNCAs to provide home care services in the future [55]. Different people could have different levels of magnitude, strength, and generalizability of self-efficacy [65]. In this study, when nurses perceive that they can effectively use mobile applications to provide home care services, they will evoke positive emotional responses toward using the mobile applications [66]. Then, the positive emotional responses promote nurses to use mobile applications [67]. Besides, self-efficacy could also produce a sense of control and agency, which leads to the use of MHNCAs according to social cognitive theory [44]. Several prior studies have shown the relationship between self-efficacy and mobile application use [68, 69]. Therefore, we hypothesize as:

  H1a: Nurses' self-efficacy positively affects their use of MHNCAs to provide home nursing care.

Outcome expectations can be defined as nurses' judgment of the likely outcomes of using MHNCAs to provide home nursing care [55]. Nurses who associate with using mobile applications would produce emotional experience from the possible expected outcomes while using [70]. Then, the emotional experience drives nurses to adopt or reject the use of mobile applications [71]. Therefore, the more positive the expected outcomes, the more nurses will use mobile applications [72]. The expected outcomes of using the mobile applications could also evoke the control and agency sense based on social cognitive theory. The higher level of the sense, the more probability of using the mobile applications [44]. Previous literature has revealed the relationship between outcome expectations and mobile application use [73, 74]. Therefore, we hypothesize as:

  H1b: Nurses' outcome expectations positively affect their use of MHNCAs to provide home care service.

Besides, nurses' self-efficacy is hypothesized to impact their outcome expectations. When nurses have the belief that they have the capability of using MHNCAs well, they would take active actions to achieve the positive outcome, then expect the positive outcomes of using MHNCAs [75]. The positive outcomes of using MHNCAs could include the good performance of providing home care service, increased income and satisfaction from patients who receive home care services, etc. Previous literature has conveyed the relationship between self-efficacy and outcome expectations like Moon and Backer [76] and Chang et al. [54]. Therefore, we hypothesize as:

  H1c: Nurses' self-efficacy positively affects their outcome expectations of using MHNCAs to provide home care service.

In social cognitive theory, sources of self-efficacy and outcome expectations are identified as mastery experiences, modeling, social persuasion, and physiological states [77]. Therefore, these sources could serve as the mechanisms for the effects of environmental and personal factors on self-efficacy and outcome expectations.

In this study, we hypothesize that organizational climate would impact nurses' self-efficacy and outcome expectations. We use organizational climate to reflect the role of organizations to which nurses belong. Organizational climate reflects members' collective perceptions of their organizations [78]. Thus, organizational climate arises from the interaction among their members and serves as the basis for members' behaviors and their understanding of organizational environments [79]. To characterize organizational climate, we decompose it into three dimensions: innovativeness, fairness, and affiliation [59]. Although these three dimensions are originally proposed in knowledge sharing context, they are also feasible to be used in nurses' MHNCA use context based on two reasons: First, MHNCAs as the information systems do have the functions to support the knowledge sharing from nurses or patients with each other. Second, since MHNCAs are used outside hospitals by nurses; the use of MHNCAs is novel for traditional hospitals and needs the support from hospitals [80]. The support from hospitals could be reflected as the trust, tolerance, and norms of hospitals. Therefore, the three dimensions of organizational climate could be applied to understand the impact of environment on nurses' MHNCA use [59].

For innovativeness, it reveals nurses' perception that their organizations encourage them to create novel solutions to their working problems and tolerate possible failure [81]. When nurses perceive that their organizations have an innovative climate, they would be encouraged and supported to use mobile applications to provide home care services since both mobile applications and home nursing care are novel [82]. Then, nurses' self-efficacy and outcome expectations would increase because organizations' support could lead to enactive mastery experiences and positive outcomes [83]. Toward fairness, it refers to nurses' perception that their organizations treat them fairly and equally in terms of procedures, distribution, or interaction [84]. Therefore, if nurses feel their organizations have a fair work culture, they will commit to their organizations and try to contribute to them [85]. Then, nurses' self-efficacy and outcome expectations would increase since their commitment to their organizations could also produce an enactive mastery experience and positive outcomes. Regarding affiliation, it can be defined as nurses' perception that their organizations encourage prosocial behaviors among their members [86]. Therefore, once nurses perceive their organizations have the affiliation climate, they may also commit to their organizations since they may receive support and help from other nurses through modeling. Then, nurses' self-efficacy and outcome expectations could increase. Summarizing the above theorizing, we hypothesize as:

  H2a: Organizational climate, characterized as innovativeness, fairness, and affiliation, affects nurses' self-efficacy positively.
  H2b: Organizational climate, characterized as innovativeness, fairness, and affiliation, affects nurses' outcome expectations positively.

Regarding the application factors, we include three factors: perceived security protection, a monetary incentive, and ease of use. Perceived security protection refers to nurses' perception that the MHNCAs, which they used to provide home care service, have taken measures to protect nurses' security in terms of transactions, services, or devices [87]. If nurses find their security requirements are satisfied by the mobile applications because of their security protection measures, they will feel little risk and trust in using them [60]. This trust, which is a mastery experience, can enhance their self-efficacy and allow them to expect favorable outcomes from using mobile applications to provide home care services [56, 88]. Therefore, we hypothesize as:

  H3a: Perceived security protection affects nurses' self-efficacy positively.
  H3b: Perceived security protection affects nurses' outcome expectations positively.

Monetary incentives are financial rewards or bonuses given by mobile application providers when nurses use mobile applications to provide home care services [89]. Monetary incentives have become common practices used by mobile application providers to attract users to use them nowadays [90]. Monetary incentives are usually provided with specific goals that require nurses to finish some tasks. These goals act as guidelines for using mobile applications and could boost nurses' self-efficacy through evoking the mastery experience [91]. Meanwhile, monetary incentives may represent the direct outcomes expected by nurses and then increase the nurses' outcome expectations. Therefore, we hypothesize as:

  H4a: Monetary incentives affect nurses' self-efficacy positively.
  H4b: Monetary incentives affect nurses' outcome expectations positively.

Regarding the ease of use, it is nurses' perception that mobile applications are easy to use to provide home care services [61]. Ease of use implies the design effectiveness of mobile applications [92]. Mobile applications, which are easy to use by nurses, are less complex and require little effort [93]. Thus, free of effort in using mobile applications could produce a highly enactive mastery experience and increase nurses' self-efficacy. Meanwhile, free of effort conveys that it would be easy to achieve the expected outcomes of using mobile applications. Therefore, we hypothesize as:

  H5a: Ease of use affects nurses' self-efficacy positively.
  H5b: Ease of use affects nurses' outcome expectations positively.

Factors in nurses' social relationships may also influence their use of mobile applications to provide home nursing care. This study focuses on two social factors: subjective norm and family support. Subjective norm is nurses' perceived social pressures from their friends, relatives, or colleagues to use mobile applications to provide home care services [94]. Subjective norm represents normative social influence and could convey the vicarious experience of using mobile applications to provide home care services to other nurses [95]. This vicarious experience of other nurses could increase the self-efficacy of the focal nurses [96]. Meanwhile, the use outcomes of other nurses could serve as an important reference for focal nurses to form their outcome expectations. Therefore, we hypothesize as:

  H6a: Subjective norm affects nurses' self-efficacy positively.
  H6b: Subjective norm affects nurses' outcome expectations positively.

Family support refers to nurses' perception of receiving care and help from their family members [97]. Support from family members is essential for nurses grappling with work–family conflicts [98]. Social support from family members can furnish nurses with the necessary resources while alleviating family burdens. [99]. Consequently, their self-efficacy in using MHNCAs will increase due to the mastery experience gained from these social resources and the alleviation of family burdens. Meanwhile, the provided social resources and the reduced family burdens could reinforce nurses' belief in their ability to successfully use mobile applications to provide home care services [100]. Therefore, we hypothesize as:

  H7a: Family support affects nurses' self-efficacy positively.
  H7b: Family support affects nurses' outcome expectations positively.

To improve the validity of our proposed research model, we also include several control variables, including age, gender, education levels, rankings, marital status, frequency of MHNCA use, working experience, and support for older adults and children.

## 2. Materials and Methods

### 2.1. Measurement Instrument

The two-wave survey is utilized to validate our research model. Compared with a cross-sectional survey, a two-wave survey has advantages in reducing the effect of common method bias and improving causal inference [101]. Therefore, using a two-wave survey could ensure the quality of our study method. Meanwhile, the survey was implemented in China based on the following reasons: First, China has the largest number of older adults in the world [102]. 4 This number is projected to reach 246 million by 2030. Thus, China has the most urgent need for home nursing care services globally. Second, China has the largest number of nursing professionals in the world [103]. This figure is expected to reach 600,000 in 2030. Thus, China would have enough home nursing care service providers. Therefore, administering our survey in China could not only study Chinese nurses but also provide implications for other countries.

To develop the measurement instrument for each construct, we adapted previously qualified instruments to suit our research context. To be specific, the items of three first-order constructs of organizational climate were adapted from Bock et al. [59]; items of perceived security protection were adapted from Kim et al. [60]; while those for monetary incentive were adapted from Zhao et al. [104]; items of ease of use were from Venkatesh and Davis [61]; items of the subjective norm were from Taylor and Todd [63]; items of family support were adapted from Procidano and Heller [62]; items of self-efficacy and outcome expectations were from Compeau et al. [105]; and items of use of mobile home care applications were from Kim et al. [60]. All the items are rated on a 7-point Likert scale. Following the guidelines of Polites et al. [106], we treated all the first-order constructs as reflective constructs and the organizational climate as the second-order formative construct. Our research plan has been submitted to and approved by the institutional review board of Tongji Medical College, Huazhong University of Science and Technology (No. 2022S181).

To ensure the measurement instrument quality, we took two main actions: First, we employed the back-translation method to ensure consistency between the English and Chinese instruments [107]. One bilingual author translated the original English instrument into Chinese, while another bilingual author back-translated the Chinese instrument into English. Then, they addressed the inconsistencies between the original English and the back-translated English versions and decided on the Chinese version of the instrument. Second, to increase the face validity, we invited five Chinese nurses and eight experts in health information systems to fill out and evaluate the Chinese instrument. The instrument was determined according to their suggestions and comments by revising and removing ambiguous and inappropriate items. The details of the measurement instrument are presented in Table 1.

### 2.2. Data Collection

To effectively administer the survey, we used the online panel services provided by Credamo,5 which provided access to nurses in China by using random sampling strategies. Credamo is a popular online survey company with a sample pool of more than 2 million respondents from diverse backgrounds in China. To ensure the data collection quality, we implemented several measures in accordance with recent methodological guidelines about online surveys [108, 109]. First, screening questions were designed to verify the respondents were nurses who had experience using mobile applications to provide home care services, such as, *how long have you been a nurse? which departments do you work in your institution? have you provided home care services by using some mobile applications?* Second, super-respondents were detected based on the completion time. Third, we set reverse-coded and attention-check questions randomly in the questionnaire to ensure response accuracy. Responses from super-respondents and respondents who failed to fill out screening, reverse-coded, and attention-check questions were excluded. Finally, high-quality respondents were invited by checking their previous answer experience, credits in Credamo, and the number of adopted answers. The quality of data collection by Credamo has also been demonstrated in previous studies of top journals [110, 111].

Our data collection had two waves in the second quarter of 2023. In the first wave, we collected data on all the independent variables (e.g., organizational climate, etc.). After 6 weeks, respondents who took part in our first wave of data collection were invited by Credamo to complete the questionnaire for the dependent variable (i.e., the use of MHNCAs). In the first wave, 836 respondents were invited, and 807 valid answers were received after data filtering. In the second wave, respondents who participated in the first-round online survey were invited, and 512 attempted the second-round survey. After cleaning up the collected data, we got 445 complete and valid answers. The demographic information of the final sample is presented in Table 2, and we can find that our sample is reasonably congruent with the population of nurses in China. Based on the analysis result of G∗power and N:q rule [112, 113], the minimum requirement for sample size is 160 and 220. Therefore, the sample size of our study exceeds these numbers.

### 2.3. Data Analysis

To analyze collected data, we use several analysis techniques to validate our hypotheses. The steps and purposes of each analysis are as follows: First, we employ SEM using partial least-squares (PLS) techniques with SmartPLS 3.3.2 software. PLS technique has the advantages of analyzing research models with second-order formative constructs and predicting dependent variables [114]. Besides, PLS is available to analyze a complex model with many variables and is used for developing theories exploratorily [115]. Therefore, it is appropriate to use PLS in this article. By following the two-step approach [116], we analyze the measurement model to check the reliability and validity of our measurement instruments and then the structural model to examine the hypothesized relationships [117]. Second, we also conducted ANN analysis to further explore the nonlinear and noncompensatory effects of the factors on nurses' use of MHNCAs. By following the guidelines from Leong et al. [118], we construct an ANN model and examine the model fit at first. Then, we implement the sentiment analysis to identify the relative importance of each predictor.

The reasons why we combine PLS-SEM and ANN are as follows: First, both nonlinear and linear, compensatory and noncompensatory effects of factors in the research model are explored through the combination of PLS-SEM and ANN. Therefore, a comprehensive view of the effects of factors is provided through combining these two techniques. Second, PLS-SEM could test the proposed hypotheses, while ANN could analyze the prediction of factors. Therefore, both the confirmative and predictive roles of factors are uncovered through combining these two techniques. In sum, the analysis results of these two techniques could complement with each other [118]. Besides, the combination of these two analysis techniques has been validated in many technology use studies [119, 120].

## 3. Results

### 3.1. Measurement Model Analysis Results

To assess the validity and reliability, we calculate the corresponding indicators according to qualified guidelines and present the results in Tables 3, 4, 5, 6 [121]. The results in Table 3 show that the values of outer loadings for each item, Cronbach's alpha, and composite reliability are all above 0.7, while the average variance extracted values for each construct are above 0.5 [122]. Therefore, the convergent validity and reliability of our measurement model are acceptable. Toward the discriminant validity, the loading values of each item are the highest for its respective latent variable in Table 4. In contrast, the correlation coefficient values between constructs are lower than the corresponding square root of the average variance extracted in Table 5. [123]. Meanwhile, all the heterotrait–monotrait ratio values are below 0.9 in Table 6 [124]. Therefore, the discriminant validity of our measurement model is confirmed. Besides, the values of variance inflation factors for every item are below 3.0, indicating that multicollinearity is insignificant [125].

Since we use the online survey method to collect data from nurses, common method bias should be tested before we examine our proposed hypotheses. Our study adopts procedural and statistical remedies by referring to classic guidelines for addressing common method bias [126]. For the procedural remedies, we used the two-wave survey to separate the measurement of dependent and independent variables, maintain the anonymity of respondents, counterbalance the order of items, and ensure the quality of measurement instruments. Regarding the statistical remedies, we used the marker variable method and chose blue attitude as the marker variable in our survey by following a relevant guideline [127]. The average correlation between blue attitude and variables in our research model was insignificant (*β* = 0.01, *p* > 0.05), showing that the issue of common method bias is not serious in this study.

### 3.2. Structural Model Analysis Results

**Figure 2:**
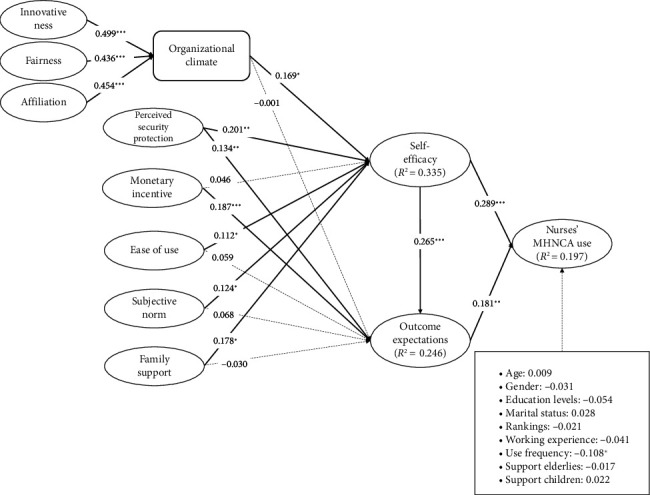
Analysis results of the structural model. Note: ^∗∗∗^*p* < 0.001, ^∗∗^*p* < 0.01, and ^∗^*p* < 0.05.

Using the PLS algorithm and bootstrapping technique with 5000 samples, we estimated the structural model by calculating path coefficients and their corresponding *p*-values for hypothesized relationships [128]. According to Figure 2, both self-efficacy and outcome expectations significantly affect nurses' use of MHNCAs positively, while self-efficacy is found to affect outcome expectations significantly. Therefore, H1a, H1b, and H1c are supported. These results imply that the two personal factors from social cognitive theory are important for nurses' use of MHNCAs. Besides, organizational climate, which is shown to be well characterized by innovativeness, fairness, and affiliation, has a significant effect on self-efficacy but not on outcome expectations. Therefore, H2a is supported, but H2b is not supported. These results show that organizational climate only partially affects personal factors. For application factors, perceived security protection significantly affects both self-efficacy and outcome expectations. The monetary incentive only significantly affects outcome expectations but not significantly affect self-efficacy, while ease of use has a significant effect on self-efficacy but not outcome expectations. Therefore, H3a, H3b, H4b, and H5a are supported, while H4a and H5b are not. These results indicate that different application factors have different effects on personal factors. Regarding social factors, subjective norm and family support only significantly affect self-efficacy but not outcome expectations. Therefore, H6a and H7a are supported, while H6b and H7b are not. These results show that social factors partially impact personal factors. Moreover, our model's predictive strength for dependent variables is acceptable [129]. Finally, except for use frequency, other control variables do not significantly affect nurses' use of MHNCAs.

### 3.3. Mediation Analysis

To detect the role of self-efficacy and outcome expectations as the underlying mechanisms, we examined their mediation effects by using the bootstrapped confidence interval test for mediation [130, 131]. The results of the test are depicted in Table 7. The results show that self-efficacy fully mediates the relationship between organizational climate and nurses' use of MHNCAs and the relationship between ease of use and nurses' use of MHNCAs, while self-efficacy partially mediates the relationship between perceived security protection and nurses' use of MHNCAs, the relationship between subjective norm and nurses' use of MHNCAs, and the relationship between family support and nurses' use of MHNCAs. Regarding outcome expectations, we find it fully mediates the relationship between self-efficacy and nurses' use of MHNCAs, partially mediates the relationship between perceived security protection and nurses' use of MHNCAs, and the relationship between monetary incentives and nurses' use of MHNCAs. Therefore, the mediation analysis results also confirm our proposed hypotheses.

### 3.4. ANN Analysis

**Figure 3:**
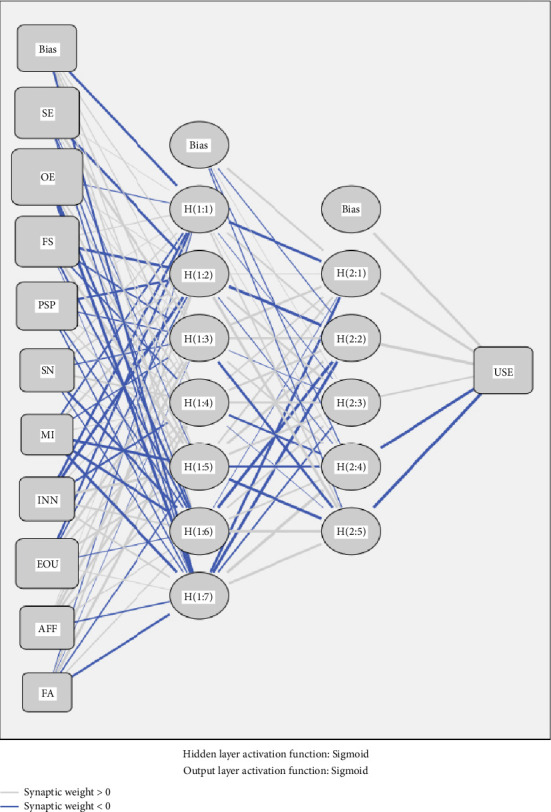
The example of the ANN model.

To explore the noncompensatory and nonlinear relationship between factors and the dependent variable, we conducted the ANN analysis following the SEM analysis by using Statistical Package for Social Science (SPSS) software, version 21. Since nurses' use of MHNCAs is our endogenous variable, we have constructed an ANN model that treats all the identified factors in SEM as the input neurons. A description of the ANN model is shown in Figure 3. In the deep-learning analysis, we have employed the feed-forward-backward-propagation (FFBP) algorithm, integrating multilayer perceptrons and sigmoid activation functions for both input and hidden layers. The hidden neurons were generated automatically. By following recent guidelines for the ANN analysis [118], we have allocated 90% of the sample data for training and 10% for testing. Besides, to avoid the risk of over-fitting, a 10-fold cross-validation process was adopted, and root-mean-squared error (RMSE) values were obtained.  Table 8 shows that the average values of RMSE for both training and testing samples are 0.124 and 0.121, respectively, which are relatively low. Therefore, the ANN model could be considered to have a good model fit and an adequate accuracy level for prediction [132].

To evaluate the predictive power of each input neuron, we have conducted a sensitive analysis to figure out their average importance and normalized importance, which could rank the predictors. The normalized importance was calculated by dividing the average importance of each predictor by the maximum average importance value. According to Table 9, the analysis results show that outcome expectation (100%) is the strongest predictor of nurses' use of MHNCAs, followed by perceived security protection (93.7%), ease of use (85.0%), family support (82.4%), self-efficacy (77.1%), innovativeness (55.0%), subjective norm (45.1%), affiliation (39.3%), fairness (38.6%), and monetary incentives (34.8%).

## 4. Discussion

This study explores the factors of a novel mobile application that nurses could use to provide home care services for patients and connect with them. Based on social cognitive theory, organizational, application, social, and personal factors are identified. Organizational, application, and social factors are hypothesized to impact nurses' use of MHNCAs through personal factors. Through a longitudinal survey, we examine the proposed hypothesized relationships. The SEM analysis results show that both self-efficacy and outcome expectations impact nurses' use of MHNCAs, while organizational, application, and social factors have differential effects on self-efficacy and outcome expectations. Specifically, organizational climate, ease of use, subjective norm, and family support affect self-efficacy significantly but not outcome expectations, while monetary incentives influence outcome expectations significantly but not self-efficacy. Only perceived security protection has significant effects on both self-efficacy and outcome expectations. Finally, the mediating roles of self-efficacy and outcome expectations are revealed.

The insignificant relationship between organizational climate and outcome expectations may be due to the fact that the organizational culture of medical institutions to which nurses belong highlights healthcare itself rather than the use of MHNCAs [133]. Regarding the relationship between monetary incentives and self-efficacy, the possible reason for this insignificant relationship is that monetary incentives do not involve the knowledge or ability to use MHNCAs. Regarding the relationship between ease of use and outcome expectations, the possible reason for this insignificant relationship is that ease of use emphasizes the interactional design of MHNCAs, not the main functionalities, and thus is not close to the outcome of MHNCA use. Considering the relationship among family support, subjective norm, and outcome expectations, the possible reasons for these insignificant relationships are that these social factors involve social relationships and resources but do not directly influence the outcome of using MHNCAs.

The ANN analysis results show that outcome expectation is the strongest predictor of nurses' use of MHNCAs. This result indicates that the outcome of using MHNCAs determines nurses' use of these applications to provide home care services. Meanwhile, perceived security protection serves as the second strongest predictor of nurses' use of MHNCAs. This result indicates that the security protection of MHNCAs should be emphasized compared with other technical and social attributes.

### 4.1. Theoretical Implications

This study could have several theoretical implications as follows:

First, novel factors of mobile health application use are explored from the novel mobile nursing applications and nurse perspective. Compared with previous literature on the mobile app usability [134, 135] and the use of mobile health applications that provide health information for patients or consumers [30, 92], this study investigates the use of mobile health applications by nurses for providing home care services to patients. Thus, this study investigates different users of mobile health applications from previous literature on mobile health applications. Meanwhile, compared with previous nursing literature on nursing or mobile nursing information system use within medical institutions [26, 136], this study focuses on the use of mobile health applications by nurses outside medical institutions. Thus, this study covers different types of mobile health applications from previous literature on nursing information systems. Therefore, since this study examines different users and different types of information systems compared with previous literature, the factors of using MHNCAs, which connect nurses with patients online, are novel for the current relevant literature on nurses' technology use and reflect the characteristics of nursing context better. Besides, the linear and nonlinear effects of these novel factors on nurses' use of MHNCAs are also determined in this paper. Therefore, novel knowledge from our study is brought to both nursing and information system disciplines.

Second, social cognitive theory is developed by being contextualized and extended in nursing context. As the overarching theory, social cognitive theory supports us in exploring the factors and underlying mechanisms of nurses' use of MHNCAs. To develop social cognitive theory, we contextualize it by decomposing the environmental factors into three categories: organizational, application, and social factors [137]. Meanwhile, we recontextualize the relationship between environmental factors and personal factors by assuming that environmental factors affect nurses' use of MHNCAs through personal factors [138]. The results of our empirical study confirm the effectiveness of our effort to contextualize social cognitive theory. Besides, the validation of the application of social cognitive theory in our study could imply that social cognitive theory could be generalized to the nursing context and the application scope of social cognitive theory is enlarged. Therefore, we extend the social cognitive theory to “nursing context and facilitate the development of social cognitive theory.”

Third, the mediating roles of self-efficacy and outcome expectations are confirmed for the nurses' use of MHNCAs. Given that mediators could help explain the effect of independent variables on dependent variables and serve as the underlying mechanisms, they are critical for theorizing [139]. Therefore, the mediating roles of self-efficacy and outcome expectations are highlighted using a bootstrapping method in our proposed research model. Specifically, self-efficacy is found to fully mediate the effect of organizational climate and ease of use on nurses' use of MHNCAs. This result shows the importance of self-efficacy in the above relationships. Meanwhile, self-efficacy partially mediates the effect of perceived security protection, subjective norm, and family support on nurses' use of MHNCAs. Moreover, outcome expectations partially mediate the effect of perceived security protection and monetary incentives on nurses' use of MHNCAs. Additionally, outcome expectations fully mediate the effect of self-efficacy on nurses' use of MHNCAs. Therefore, the mediating roles of self-efficacy and outcome expectations are revealed for the above effects and confirmed for the nursing context [67, 71].

To conclude, our study not only tests the social cognitive theory in a new context, that is, nurses' use of MHNCA context, but also adds new factors to the environmental factors of social cognitive theory. Therefore, according to recent guidelines, our study gives adequate theoretical contributions [140].

### 4.2. Implications for Nursing Management

Besides the theoretical implications, implications for nurse community, nursing managers, and nursing educators are also provided as follows:

First, nursing managers could facilitate the formation of the organizational culture, which support nurses' use of MHNCAs. To facilitate nurses' use of MHNCAs, the medical institutions to which nurses belong should provide a supportive climate. Nursing managers could foster an innovative organizational climate by providing financial and nonfinancial rewards for nurses' effective innovation. Additionally, they can promote a supportive organizational culture through the development of fair norms or policies and encouraging an affiliative organizational climate achieved by enhancing nurses' sense of identification with their medical institution. Therefore, nursing managers could take multiple measures to produce a supportive organizational climate.

Second, nursing managers should emphasize the design of MHNCAs. Three application factors, including perceived security protection, monetary incentives, and ease of use, influence nurses' use of MHNCAs. Therefore, nursing managers should choose the MHNCAs that protect the security of nurses' private information and their safety by using encryption techniques or alarm systems, providing financial rewards to nurses' use of MHNCAs based on rules that are reasonable, and reducing operating requirements to make the use of MHNCAs be easy [141].

Third, nurses could seek support from their families and follow subjective norm. Two social factors, family support and subjective norm, impact nurses' use of MHNCAs. Family support and subjective norm reflect that the social relationships are important to nurses. Based on these results, nurses should pay attention to the opinions of family members and their peers. The full communication and interaction between nurses and their families/peers should be taken before they use MHNCAs to provide home care services.

Finally, the education and training of nurses about using MHNCAs effectively should be provided. Nursing educators could provide ongoing education to nurses about how to understand and use MHNCAs effectively [142]. The education could include explaining the functions of MHNCAs, presenting the different operation ways of the functions of MHNCAs, listing the best practice of using MHNCAs in nurses' daily work, and conveying the knowledge and skills of nurses for using MHNCAs. The ongoing education should also evaluate the learning outcomes of nurses by adopting multiple examining methods.

### 4.3. Limitations and Future Research Directions

Although theoretical and practical implications are given in this study, limitations still exist, and future research directions are suggested based on these limitations.

First, other factors could be explored based on other theoretical perspectives. Although all the proposed factors are shown to impact self-efficacy or outcome expectations significantly, some effects, such as the effect of organizational climate on outcome expectations, remain insignificant. Meanwhile, the explained variance of dependent variables, including self-efficacy, outcome expectations, and nurses' use of MHNCAs, is not very high, indicating that other factors could be included to better explain the variance of dependent variables. Therefore, future studies could consider other factors from other theoretical perspectives.

Second, other mediators and moderators could be investigated. Self-efficacy and outcome expectations have been confirmed as key underlying mechanisms through mediating analysis. However, some of the proposed relationships are not fully mediated by these two mediators, like the relationship between family support and nurses' use of MHNCAs or the relationship between perceived security protection and nurses' use of MHNCAs. Besides, other moderators that convey the boundary conditions of exploring relationships in this study should be considered. Therefore, future studies could explore other factors mediating and moderating the relationships between nurses' use of MHNCAs and its factors.

Third, our proposed model could be validated in other countries. Given the cultural differences among countries, the effects of the factors in our research model may vary. For example, nurses in China, with its collectivistic culture, may respond differently to family and peer influence than those in the United States with an individualistic culture [143]. Therefore, the effect of family support and subjective norm for nurses in China would differ from those in the United States. Therefore, future studies could investigate nurses' use of MHNCAs in other countries.

Fourth, other data collection methods could be applied. In this study, we adopt the online panel service to collect data. Although several measures have been taken to ensure the quality of this data collection method, potential biases such as ignoring less technologically resilient groups may still exist. Therefore, future studies could adopt other data collection methods such as multicenter offline survey or field experiment, which could overcome the potential biases of online survey. Besides, a mixed-method design, which combines online survey and other research methods, could also be used to solve the potential biases.

## 5. Conclusion

This study investigates the use of MHNCAs, a novel mobile health application designed to connect nurses with patients from a novel perspective: the nurses' perspective. Grounded on social cognitive theory, we establish a research model to provide a complete theoretical framework to understand nurses' MHNCA use. By analyzing data from nurses who used MHNCAs, we find that environmental factors, including organizational, application, and social factors, impact nurses' use of MHNCAs, mediated by their personal cognitive factors. Besides, the outcome expectations of nurses are found to be the most powerful factor in nurses' use of MHNCAs. Then, our study not only tests and validates the applicability of social cognitive theory in nursing context but also enriches the theory by incorporating novel and context-specific factors. Furthermore, our study uncovers the underlying mechanisms for the effects of these factors. Therefore, we make significant theoretical contributions to the evolution of social cognitive theory and offer novel insights into the previous literature on nurse technology use. Additionally, our findings can benefit nursing managers, nursing educators, and the broader nursing community by informing the design of MHNCAs tailored for nurses, supporting the uptake of MHNCAs, and enhancing the training and education related to nurses' technology use. Consequently, our study underscores the cutting-edge advancements in mobile health applications within the digital realm and the nursing context, highlighting the importance of these technologies in contemporary nursing practices.

## Figures and Tables

**Table 1:** Measurement instrument.

Constructs and sources	Items
Innovativeness [59]	• My department encourages suggesting ideas for providing nursing services • My department puts much value on providing nursing services
Affiliation [59]	• Members in my department keep close ties with each other • Members in my department have a feeling of ‘one team'
Fairness [59]	• Objectives that are given to me by my superiors are reasonable • My superiors do not show favoritism to anyone
Perceived security protection [60]	• This APP implements security measures to protect nurses • This APP ensures that nurses' personal and transaction information is protected from accidently being altered or destroyed during a transmission • I feel safe in taking orders on this APP
Monetary incentives [104]	• Using this APP brings me financial benefits • Using this APP can improve my economic situation
Ease of use [61]	• Learning to use this APP is easy for me • My using of this APP to take nursing tasks is clear
Subjective norm [63]	• People who influence my behavior wanted me to use this APP • People I know influence my use of this APP
Family support [62]	• My family give me the support I need • I get good ideas about how to take nursing tasks in this APP from my family • Members of my family could help me solve problems about taking nursing tasks through this APP
Self-efficacy [105]	• I have confidence in my ability to provide nursing services through this APP • I have confidence in my ability to cope with problems in providing nursing services through this APP • I can use this app to provide nursing services
Outcome expectations [105]	• If I use this APP, I will increase my sense of accomplishment • If I use this APP, I will enhance specialized skills • If I use this APP, my co-workers will perceive me as competent
Nurses' use of MHNCAs [60]	• I use this APP • I recommend this APP to my friends • I continue using this APP in the foreseeable future

**Table 2:** Demographic information.

Variables	Number (*n* = 445)	Percentages (%)
Gender		
Male	119	26.7
Female	326	73.3
Age		
< 35	351	78.9
35–54	92	20.7
> 55	2	0.4
Marital status		
Unmarried	113	25.4
Married	332	74.6
Education level		
Some college or less	6	1.3
Bachelor's degree	403	90.6
Graduate degree	36	8.1
MHNCAs usage frequency		
Once a day	253	56.9
Once a week	184	41.3
Once a month	7	1.6
Once half a year or above	1	0.2
Number of elderlies supported		
0	40	9.0
1	81	18.2
2	259	58.2
3 or above	65	14.6
Departments		
Internal medicine	68	15.3
Surgery	131	29.4
Gynecology and obstetrics	80	18.0
Pediatrics	89	20.0
Otorhinolaryngology	19	4.3
Oncology	20	4.5
General	22	4.9
Emergency	3	0.7
Others	13	2.9
Rankings		
Nurse	246	55.3
Senior nurse	127	28.5
Nurse supervisor	64	14.4
Vice chief nurse or above	8	1.8
Working experience		
< 5 years	206	46.3
6–15 years	228	51.2
> 16 years	11	2.5
Number of children supported		
0	125	28.1
1	268	60.3
2	50	11.2
3 or above	2	0.4

**Table 3:** Convergent validity and reliability.

Constructs	Outer loadings		Variance inflation factors	Composite reliability	Cronbach's alpha	Average variance extracted
Innovativeness	INN1	0.901	1.679	0.900	0.777	0.818
	INN2	0.908	1.679			

Affiliation	AFF1	0.907	1.708	0.902	0.783	0.822
	AFF2	0.906	1.708			

Fairness	FA1	0.899	1.617	0.894	0.764	0.809
	FA2	0.900	1.617			

Perceived security protection	PSP1	0.834	1.536	0.863	0.763	0.678
	PSP2	0.821	1.651			
	PSP3	0.814	1.49			

Monetary incentives	MI1	0.873	1.422	0.872	0.705	0.772
	MI2	0.884	1.422			

Ease of use	EOU1	0.937	2.249	0.932	0.854	0.873
	EOU2	0.931	2.249			

Family support	FS1	0.793	1.535	0.873	0.78	0.696
	FS2	0.882	1.956			
	FS3	0.825	1.612			

Subjective norm	SN1	0.913	1.61	0.893	0.762	0.807
	SN2	0.883	1.61			

Self-efficacy	SE1	0.799	1.366	0.849	0.735	0.653
	SE2	0.793	1.49			
	SE3	0.832	1.554			

Outcome expectation	OE1	0.821	1.571	0.854	0.745	0.661
	OE2	0.805	1.488			
	OE3	0.814	1.421			

Nurses' use of MHNCAs	USE1	0.845	1.65	0.858	0.751	0.669
	USE2	0.766	1.345			
	USE3	0.841	1.697			

**Table 4:** Loadings and cross-loadings.

Constructs	INN	AFF	FA	PSP	MI	EOU	FS	SN	SE	OE	USE
INN1	**0.901**	0.256	0.287	0.381	0.288	0.178	0.317	0.298	0.315	0.179	0.259
INN2	**0.908**	0.276	0.317	0.325	0.198	0.125	0.298	0.211	0.27	0.19	0.259
AFF1	0.289	**0.907**	0.148	0.335	0.231	0.268	0.251	0.2	0.312	0.155	0.292
AFF2	0.244	**0.906**	0.197	0.241	0.145	0.235	0.248	0.14	0.267	0.109	0.28
FA1	0.303	0.167	**0.899**	0.273	0.219	0.229	0.325	0.221	0.274	0.2	0.249
FA2	0.298	0.175	**0.9**	0.294	0.23	0.195	0.306	0.241	0.269	0.215	0.23
PSP1	0.296	0.247	0.184	**0.834**	0.382	0.157	0.333	0.286	0.415	0.32	0.292
PSP2	0.361	0.265	0.264	**0.821**	0.392	0.163	0.387	0.315	0.326	0.282	0.296
PSP3	0.313	0.274	0.336	**0.814**	0.436	0.216	0.414	0.347	0.382	0.314	0.337
MI1	0.244	0.188	0.234	0.414	**0.873**	0.124	0.323	0.217	0.281	0.288	0.272
MI2	0.227	0.176	0.205	0.446	**0.884**	0.052	0.309	0.253	0.265	0.324	0.214
EOU1	0.157	0.253	0.196	0.214	0.057	**0.937**	0.208	0.226	0.273	0.179	0.305
EOU2	0.155	0.266	0.246	0.192	0.13	**0.931**	0.239	0.196	0.257	0.176	0.307
FS1	0.195	0.204	0.178	0.371	0.225	0.2	**0.793**	0.222	0.344	0.178	0.245
FS2	0.343	0.269	0.358	0.384	0.339	0.212	**0.882**	0.351	0.394	0.202	0.311
FS3	0.302	0.214	0.33	0.39	0.328	0.187	**0.825**	0.329	0.349	0.25	0.316
SN1	0.258	0.215	0.244	0.391	0.244	0.205	0.368	**0.913**	0.322	0.288	0.292
SN2	0.245	0.116	0.217	0.292	0.237	0.201	0.28	**0.883**	0.328	0.186	0.203
SE1	0.267	0.231	0.181	0.414	0.238	0.236	0.349	0.301	**0.799**	0.359	0.357
SE2	0.236	0.303	0.278	0.328	0.241	0.195	0.334	0.242	**0.793**	0.312	0.273
SE3	0.277	0.246	0.278	0.363	0.274	0.253	0.37	0.329	**0.832**	0.325	0.308
OE1	0.168	0.099	0.191	0.277	0.284	0.122	0.209	0.182	0.332	**0.821**	0.255
OE2	0.182	0.102	0.192	0.305	0.234	0.176	0.197	0.224	0.354	**0.805**	0.256
OE3	0.15	0.152	0.18	0.323	0.328	0.164	0.21	0.243	0.32	**0.814**	0.257
USE1	0.244	0.235	0.187	0.3	0.208	0.273	0.217	0.196	0.339	0.271	**0.845**
USE2	0.261	0.287	0.268	0.335	0.267	0.233	0.361	0.263	0.286	0.275	**0.766**
USE3	0.198	0.255	0.202	0.286	0.204	0.297	0.288	0.229	0.327	0.226	**0.841**

*Note:* INN = innovativeness, AFF = affiliation, and FA = fairness. The bold values are the loading values for each item.

Abbreviations: EOU = ease of use, FS = family support, MI = monetary incentive, OE = outcome expectation, PSP = perceived security protection, SE = self-efficacy, SN = subjective norm, and USE = nurses' use of MHNCAs.

**Table 5:** Correlations among constructs.

Constructs	INN	AFF	FA	PSP	MI	EOU	FS	SN	SE	OE	USE
INN	**0.904**										
AFF	0.294	**0.907**									
FA	0.334	0.19	**0.899**								
PSP	0.39	0.318	0.315	**0.823**							
MI	0.268	0.207	0.25	0.49	**0.879**						
EOU	0.167	0.277	0.236	0.217	0.099	**0.934**					
FS	0.339	0.276	0.351	0.457	0.359	0.239	**0.834**				
SN	0.28	0.188	0.257	0.383	0.268	0.226	0.364	**0.898**			
SE	0.323	0.319	0.302	0.458	0.311	0.284	0.435	0.361	**0.808**		
OE	0.204	0.146	0.23	0.372	0.349	0.19	0.252	0.267	0.412	**0.813**	
USE	0.286	0.315	0.266	0.375	0.276	0.327	0.35	0.279	0.389	0.315	**0.818**

*Note:* INN = innovativeness, AFF = affiliation, and FA = fairness. The bold diagonal values represent the square root of the AVE for the corresponding constructs.

Abbreviations: EOU = ease of use, FS = family support, MI = monetary incentive, OE = outcome expectation, PSP = perceived security protection, SE = self-efficacy, SN = subjective norm, and USE = nurses' use of MHNCAs.

**Table 6:** Heterotrait–monotrait ratio (HTMT).

Constructs	INN	AFF	FA	PSP	MI	EOU	FS	SN	SE	OE	USE
INN											
AFF	0.377										
FA	0.433	0.246									
PSP	0.51	0.411	0.415								
MI	0.363	0.279	0.341	0.667							
EOU	0.206	0.339	0.293	0.268	0.13						
FS	0.432	0.351	0.449	0.595	0.481	0.294					
SN	0.365	0.238	0.336	0.498	0.365	0.28	0.464				
SE	0.426	0.424	0.406	0.604	0.431	0.356	0.573	0.481			
OE	0.269	0.189	0.306	0.49	0.478	0.238	0.33	0.348	0.556		
USE	0.375	0.413	0.354	0.496	0.382	0.408	0.459	0.367	0.52	0.421	

*Note:* INN = innovativeness, AFF = affiliation, and FA = fairness.

Abbreviations: EOU = ease of use, FS = family support, MI = monetary incentive, OE = outcome expectation, PSP = perceived security protection, SE = self-efficacy, SN = subjective norm, and USE = nurses' use of MHNCA.

**Table 7:** Mediation test.

Proposed relationship	Indirect effect			Direct effect			Mediation type
	2.5% lower bound	97.5% upper bound	Include zero?	2.5% lower bound	97.5% upper bound	Include zero?	
OC ⟶ SE ⟶ USE	0.008	0.121	No	0.117	0.270	No	Full
PSP ⟶ SE ⟶ USE	0.021	0.108	No	−0.013	0.205	Yes	Partial
MI⟶SE⟶USE	−0.016	0.046	Yes	−0.050	0.144	Yes	None
EOU ⟶ SE ⟶ USE	0.007	0.073	No	0.062	0.261	No	Full
SN ⟶ SE ⟶ USE	0.003	0.074	No	−0.053	0.135	Yes	Partial
FS ⟶ SE ⟶ USE	0.011	0.106	No	−0.023	0.217	Yes	Partial
OC ⟶ OE ⟶ USE	−0.014	0.048	Yes	0.117	0.270	No	None
PSP ⟶ OE ⟶ USE	0.007	0.072	No	−0.013	0.205	Yes	Partial
MI ⟶ OE ⟶ USE	0.012	0.068	No	−0.050	0.144	Yes	Partial
EOU ⟶ OE ⟶ USE	0.000	0.041	Yes	0.062	0.261	No	None
SN ⟶ OE ⟶ USE	−0.004	0.047	Yes	−0.053	0.135	Yes	None
FS ⟶ OE ⟶ USE	−0.026	0.030	Yes	−0.023	0.217	Yes	None
SE ⟶ OE ⟶ USE	0.017	0.097	No	0.187	0.391	No	Full

*Note:* USE = MHNCA use.

Abbreviations: EOU = ease of use, FS = family support, MI = monetary incentive, OC = organizational climate, OE = outcome expectations, PSP = perceived security protection, SE = self-efficacy, and SN = subjective norm.

**Table 8:** RMSE values for training and testing of ANN.

Networks	Training			Testing		
	N	SSE	RMSE	Number	SSE	RMSE
ANN1	395	5.538	0.118	50	0.790	0.126
ANN2	400	6.088	0.123	45	0.766	0.130
ANN3	389	6.269	0.127	47	0.551	0.108
ANN4	387	6.486	0.129	58	0.806	0.118
ANN5	391	7.220	0.136	54	0.786	0.121
ANN6	403	5.726	0.119	42	0.694	0.129
ANN7	395	6.081	0.124	50	0.737	0.121
ANN8	400	6.098	0.123	45	0.833	0.136
ANN9	396	5.691	0.120	49	0.551	0.106
ANN10	402	5.366	0.116	43	0.575	0.116
Mean	395.8	6.056	0.124	48.3	0.709	0.121
Standard deviation	5.514	5.535	0.006	4.990	0.110	0.010

*Note: N* = sample size.

Abbreviations: RMSE = root-mean-square of errors and SSE = sum square of errors.

**Table 9:** Sensitive analysis of the ANN model.

Variables	Average importance	Normalized importance (%)	Ranking
SE	0.118	77.1	5
OE	0.154	100.0	1
FS	0.127	82.4	4
PSP	0.144	93.7	2
SN	0.069	45.1	7
MI	0.053	34.8	10
INN	0.085	55.0	6
EOU	0.130	85.0	3
AFF	0.060	39.3	8
FA	0.059	38.6	9

*Note:* INN = innovativeness, AFF = affiliation, and FA = fairness.

Abbreviations: EOU = ease of use, FS = family support; MI = monetary incentive, OE = outcome expectation, PSP = perceived security protection, SE = self-efficacy, and SN = subjective norm.

## Data Availability

The data that support the findings of this study are available on request from the corresponding author. The data are not publicly available due to privacy or ethical restrictions.

## Endnotes

^1^WHO is the abbreviation of the World Health Organization.

^2^We use “home nursing care” to represent nursing service in the whole home care services.

^3^The data are from https://www.goldnurse.com/.

^4^UN is the abbreviation of the United Nations.

^5^The URL is https://www.credamo.com/.
